# Baseline factors relating to depressive symptoms at one year postoperative in patients with diffuse glioma

**DOI:** 10.1093/nop/npae085

**Published:** 2024-09-20

**Authors:** Vera Belgers, Anders Tolver, Martin Klein, Linda Douw, Johanna M Niers, Karin Piil, Philip C de Witt Hamer

**Affiliations:** Amsterdam UMC location Vrije Universiteit Amsterdam, Neurology, 1081 HV Amsterdam, The Netherlands; Cancer Center Amsterdam, Brain Tumor Center, Amsterdam, The Netherlands; Statistics and Data Analysis, Danish Cancer Institute, DK-2100 Copenhagen, Denmark; Data Science Lab, Department of Mathematical Sciences, University of Copenhagen, DK-2100, Copenhagen, Denmark; Amsterdam UMC location Vrije Universiteit Amsterdam, Medical Psychology, 1081 HV Amsterdam, The Netherlands; Cancer Center Amsterdam, Brain Tumor Center, Amsterdam, The Netherlands; Amsterdam UMC location Vrije Universiteit Amsterdam, Anatomy and Neurosciences, 1081 HV Amsterdam, The Netherlands; Cancer Center Amsterdam, Brain Tumor Center, Amsterdam, The Netherlands; Amsterdam UMC location Vrije Universiteit Amsterdam, Neurology, 1081 HV Amsterdam, The Netherlands; Cancer Center Amsterdam, Brain Tumor Center, Amsterdam, The Netherlands; Department of People and Technology, Roskilde University (K.P.); Department of Oncology, Rigshospitalet, 2100, Copenhagen, Denmark; Cancer Center Amsterdam, Brain Tumor Center, Amsterdam, The Netherlands; Amsterdam UMC location Vrije Universiteit Amsterdam, Neurosurgery, 1081 HV Amsterdam, The Netherlands

**Keywords:** aphasia, disability and health, depression, fatigue, international classification of functioning, quality of life

## Abstract

**Background:**

Depressive symptoms are common in patients with diffuse glioma, potentially reducing their quality of life. Understanding baseline factors associated with the development of depressive symptoms is important for psychoeducation and early intervention. This study investigates the associations of baseline patient- and tumor-related characteristics and depressive symptoms 1 year after surgery.

**Methods:**

We combined retrospective longitudinal datasets from Amsterdam UMC and Rigshospitalet Copenhagen. Several characteristics of patients and tumors were retrieved, in particular items of their mood and functioning status. Depression instruments were harmonized to the Patient-Reported Outcomes Measurement Information System Depression scale through previously developed item response theory. Functioning items were harmonized to the International Classification of Functioning, Disability, and Health (ICF) domains using linking methods published previously. We analyzed the associations of 25 baseline factors with depressive symptoms one year after surgery by multivariable stepwise backward linear regression models and verified model robustness using best subset selection.

**Results:**

We included 118 patients with diffuse glioma with a mean age of 48 years and a glioblastoma in 29%. Baseline depressive symptoms, lower ICF energy, and impaired ICF language functioning were associated with more depressive symptoms at follow-up in the multivariable model (R-squared: 0.379).

**Conclusions:**

We identified 3 key baseline factors associated with depressive symptoms one year after surgery. Clinically, our findings contribute to the comprehension of predictive factors for depressive symptoms, aiding healthcare providers and patients in understanding and possibly allowing for early intervention.

Patients diagnosed with diffuse glioma (World Health Organization [WHO] grades 2–4) often face multiple challenges, not only physical and cognitive burdens but also emotional distress.^1^ Notably, depressive symptoms are common in patients with glioma, ranging from 16% to 41%, and are associated with worse health-related quality of life (HRQoL) and shorter survival.^2–4^ A recent study involving 74 brain tumor patients across different disease phases showed depressive symptoms related to antidepressant use, a history of stressor/trauma-related disorder, and HRQoL.^5^ Other factors linked to depressive symptoms include cognition, time since diagnosis, function, and tumor volume, though studies yield inconsistent and sometimes conflicting results.^6^ Another recent review identified Karnofsky Performance Status (KPS), fatigue, and certain medications as related to depressive symptoms, and concluded that high-quality evidence is limited.^2^ The challenges faced by patients after a glioma diagnosis are complex and can be assessed through a biopsychosocial model that incorporates various aspects of a patient’s life.^1^ Little is known about how depressive symptoms in the long term relate to baseline psychological, physical, activity, and participation functions.

To integrate the assessment of these biopsychosocial functions, the WHO has devised the International Classification of Functioning, Disability, and Health (ICF) Framework, capturing several health domains: body functions, body structure, activity, participation, and environment.^7^ It assesses a person’s complete functioning rather than specific factors, to capture the full impact on a person’s life and their ability to function in daily activities. For example, a patient with visual impairment (body function) from a glioma might have trouble reading (activity and participation) and receive assistance from their family (environment). Several instruments are available to assess a patient’s functional status. Typical instruments in use for patients with brain tumors are the EORTC brain cancer module (EORTC QLQ-BN20; BN20),^8^ the brain subscale of the functional assessment of cancer therapy (FACT-Br),^9^ or the 36-item short form health survey (SF-36).^10^ To enhance comparison between functional assessments, linking rules have been established to link various instruments’ items that represent the same underlying meaningful ICF health domains.^11^ For instance, the BN20, the FACT-Br, and the SF-36 contain items that link to body functions, activity, and participation, whereas only the FACT-Br has items linking to the environment.^12^

This study aimed to determine the risk factors at baseline for depressive symptoms one year postoperative in patients with diffuse glioma. We considered both tumor and patient characteristics, including individual ICF domains (functioning factors), and latent profiles (LPA). The LPA was used to integrate multiple functioning domains, providing a holistic view rather than assessing functions in isolation. We focused on baseline factors as patients with a glioma typically undergo surgery, rendering the perioperative phase a universal, distinct early phase that allows for an opportunity for early identification of risk factors. Early recognition of individuals at risk for depressive symptoms in the long term, would ideally enable timely psychoeducation, interventions targeting modifiable risk factors, and support tailored to unmodifiable risk factors. This could potentially reduce the long-term impact on patients’ mental well-being. Nevertheless, limited healthcare resources and the potential lack of motivation of patients for support programs to prevent future depressive symptoms should be addressed.

## Methods

### Patients

In this retrospective longitudinal study, we combined cohorts from Amsterdam UMC and Rigshospitalet Copenhagen with patients treated between 2007 and 2023, aged ≥18 years, had a histopathological diagnosis of a supratentorial diffuse glioma of WHO 2016 grade II–IV, and had depressive symptom measurements both at baseline and a year postoperatively. Patients could be newly diagnosed or have a repeated resection. We incorporated one baseline measurement and another approximately 1 year postoperatively for each patient. If a patient had multiple measurements, we included the measurement closest to surgery within a range of 6 months before to 6 weeks after surgery and the measurement closest to 1 year post-surgery within a range of 6 months to 2 years after surgery.

Reports from either cohort have been previously published with different research questions.^13–15^ Study protocols were approved by local medical ethical boards.

### Outcomes

The outcome was depressive symptoms a year after surgery. For the Amsterdam cohort, the Center for Epidemiologic Studies Depression Scale (CES-D) was used for assessing depressive symptoms,^16^ while the Copenhagen cohort applied the Hospital Anxiety and Depression Scale—Depression (HADS-D).^17^ To harmonize these instruments, we converted them to the patient-reported outcomes measurement information system (PROMIS) depression scale using the linking tables provided through the “PROsetta Stone” project.^18,19^ This project has the goal of linking different instruments measuring the same underlying construct, using item response theory with fixed parameter calibration. The PROMIS-Depression scale ranges from 0 to 100 with a mean of 50 and a standard deviation (SD) of 10, with higher scores representing more depressive symptoms.^20^ This continuous score was used for the analyses; however, cutoffs based on the oncology population were used to gauge the prevalence of depressive symptoms in our population: A PROMIS-Depression score of <52.5 was considered no depressive symptoms, a score between 52.5 and 62.5 as at least mild depressive symptoms, and ≥62.5 indicated at least moderate symptoms.^20^

We evaluated 25 patient- and tumor-related characteristics as potential risk factors, see both Table 1 and Supplementary Table 1. Patient-related characteristics consisted of baseline age, sex, education level, performance status, depressive symptoms, and ICF health domains.

**Table 1.** Baseline Characteristics Excluding ICF Domains

	Overall (*N* = 118)	Amsterdam (*N* = 85)	Copenhagen (*N* = 33)	*P*-value^a^
Age				
Mean (SD)	48.0 (14.4)	43.8 (12.1)	58.7 (14.5)	<.001
Sex				
Male	76 (64.4%)	54 (63.5%)	22 (66.7%)	.832
Female	42 (35.6%)	31 (36.5%)	11 (33.3%)	
Education level				
High	55 (46.6%)	40 (47.1%)	15 (45.5%)	.085
Medium	35 (29.7%)	21 (24.7%)	14 (42.4%)	
Low	28 (23.7%)	24 (28.2%)	4 (12.1%)	
Performance status				
Fully active	77 (65.3%)	57 (67.1%)	20 (60.6%)	.229
Minor restrictions	34 (28.8%)	25 (29.4%)	9 (27.3%)	
Limited activity	7 (5.9%)	3 (3.5%)	4 (12.1%)	
Depressive symptoms at baseline				
Median [Min, Max]	53.4 [34.5, 67.0]	52.6 [34.5, 67.0]	57.1 [35.8, 64.3]	.016
LPA functioning class				
High	51 (43.2%)	39 (45.9%)	12 (36.4%)	.129
Moderate	48 (40.7%)	36 (42.4%)	12 (36.4%)	
Low	19 (16.1%)	10 (11.8%)	9 (27.3%)	
Tumor location				
Frontal lobe	63 (53.4%)	52 (61.2%)	11 (33.3%)	.008
Other than frontal lobe	55 (46.6%)	33 (38.8%)	22 (66.7%)	
Tumor side				
Left	62 (52.5%)	45 (52.9%)	17 (51.5%)	.11
Right	52 (44.1%)	39 (45.9%)	13 (39.4%)	
Bilateral	4 (3.4%)	1 (1.2%)	3 (9.1%)	
Tumor histology^b^				
Astrocytoma	43 (36.4%)	37 (43.5%)	6 (18.2%)	<.001
Oligodendroglioma	40 (33.9%)	39 (45.3%)	1 (3.0%)	
Glioblastoma	34 (28.8%)	9 (10.5%)	25 (75.8%)	
Other	1 (0.8%)	0 (0%)	1 (3.0%)	
Tumor grade^b^				
II	54 (45.8%)	54 (63.5%)	0 (0%)	<.001
III	29 (24.6%)	22 (25.9%)	7 (21.2%)	
IV	35 (29.7%)	9 (10.6%)	26 (78.8%)	

^a^Difference between cohorts, as tested by Mann–Whitney *U* test for continuous data and Fishers’ exact test for categorical data.

^b^According to the WHO 2016 classification.

### Statistical Analyses

We classified education level as low, medium, and high according to the Verhage scale for the Amsterdam cohort and we matched this to the Danish system for the Copenhagen cohort: completed ninth grade was considered low education, completed vocational education, upper secondary education, and short-cycle higher education were considered medium education level, and medium-length and long-cycle higher education were considered high education level.^21^ For performance status, a KPS of 90 or 100 for both cohorts or an Eastern Cooperative Oncology Group Performance Status (ECOG) of zero for the Copenhagen cohort was considered fully active, a KPS of 70–80 or an ECOG one as minor restrictions in performance and everything else as limited activity.^22,23^

For ICF domains, the EORTC QLQ-BN20^8^ (Amsterdam), 36-Item Short Form Survey^10^ (SF-36; Amsterdam), and FACT-Br^9^ (Copenhagen) were used to create scores ranging from 0 to 100, with higher scores representing better functioning. The EORTC QLQ-BN20 is a brain tumor symptom-specific supplementary module of the EORTC QLQ-C30 health-related quality of life questionnaire. Its items range from 0 to 100, for multi-item subscales the items are averaged. Higher scores on the EORTC QLQ-BN20 represent worse functioning. Therefore, for this study, scores were reversed. The SF-36 is a generic health-related quality of life questionnaire. Items are scaled to range from 0 to 100, higher reflects better functioning and subscales consist of averaged item scores. The FACT-Br is the brain-specific addition to the functional assessment of cancer therapy–general scale (FACT-G). Items are scored and then linearly transformed, and for subscales, the items are averaged. For harmonization with the EORTC QLQ-BN20 and SF-36, we multiplied the items by 25 to obtain scores from 0 to 100 in concordance with the EORTC QLQ-BN20 and SF-36. The items of these instruments have been systematically linked to ICF domains, and the authors^12^ provided us with their results on an item level. See Supplementary Material for more background on composing the ICF domains. Tumor-related characteristics consisted of tumor location (frontal or non-frontal), tumor side (left, right, or bilateral), histology, and tumor grade. In addition to establishing the baseline ICF functioning domains, we conducted a latent profile analysis (LPA) to organize these domains into distinct categories for individuals. This offers a comprehensive perspective on patients’ overall functioning and could potentially be more closely linked to depressive symptoms than individual ICF functioning domains.

Non-normal distributions of baseline characteristics were represented by the median and range and normal distributions by the mean and SD for each cohort. We analyzed the differences between cohorts by using a Mann–Whitney *U* test for continuous data and a Fishers’ exact test for categorical data and then combined the cohorts for all further analyses. We did our analyses in R v4.2.1, and *P*-values <.05 were considered statistically significant.

We only included complete cases, ie, those patients who had both baseline and post-surgery assessments, along with all 25 baseline factors. To evaluate the association between the potential risk factors and depressive symptoms at follow-up, we performed forward selection through univariable linear regression analyses for each of the 25 factors and factors with a *P*-value of <.1 were considered for subsequent analyses. With these selected factors, we performed multivariable linear regression with a stepwise backward selection. We chose the best-fitting regression model based on the lowest Akaike Information Criterion.

To examine the multicollinearity of the continuous baseline variables, we constructed a matrix of Spearman’s correlations. Correlation coefficients of 0.00–0.10 were considered negligible, 0.10–0.39 weak, 0.40–0.69 moderate, 0.70–0.89 strong, and 0.90–1.00 very strong.

Next, to investigate the robustness of our findings, we repeated the stepwise backward selection without the LPA classes. Additionally, we performed the best subset selection on the variables that were selected through forward selection using the “leaps” R package. Best subset selection assesses all possible subsets of variables and quantifies the best model for each size of the input set, rather than assessing the best models only for the decreasing number of variables through the stepwise backward selection. This method can be used to confirm that the backward selection procedure did not miss a better model with a different combination of variables that were previously removed in the backward regression.

## Results

### Patients

**Figure 1.**
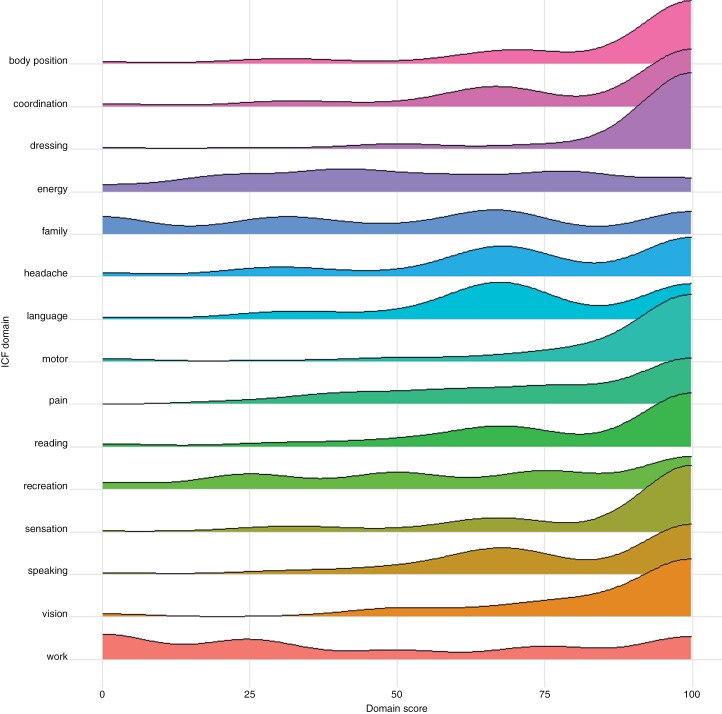
Ridge plot of baseline ICF domains. Higher scores reflect better functioning.

We identified 130 eligible patients, 12 patients were excluded due to missing data (sex, performance status, tumor side, or ICF domains), resulting in 118 patients in total: 85 from Amsterdam and 33 from Copenhagen. The mean age was 48 years (SD 14.4), and 64.4% was male. In the Copenhagen cohort, patients were older, had a glioblastoma diagnosis more often and locations other than the frontal lobe were more frequent compared to the Amsterdam cohort, as listed in Table 1. See Figure 1 for the distribution of the patients’ ICF domain scores. For all further analyses, we assessed depressive symptoms continuously. To better understand the population, however, we also calculated the percentage of people with depressive symptoms at follow-up: 41% had no depressive symptoms, 50% had at least mild depressive symptoms, and 9% had at least moderate depressive symptoms.

### Regression Analyses for Potential Risk Factors Related With Depressive Symptoms at Follow-up

As a first step, we conducted univariable regression analyses to determine which variables to include in the forward selection for the multivariable model. This forward selection identified the following factors to be added to the backward selection step: medium education, lower performance status, higher baseline depressive scores, and lower ICF functions regarding energy, language, pain, motor function, coordination, sensation, reading, body position, family, work, recreation, lower vision, speaking function, and patients belonging to the moderate or low functioning LPA class (Supplementary Table 1).

**Figure 2.**
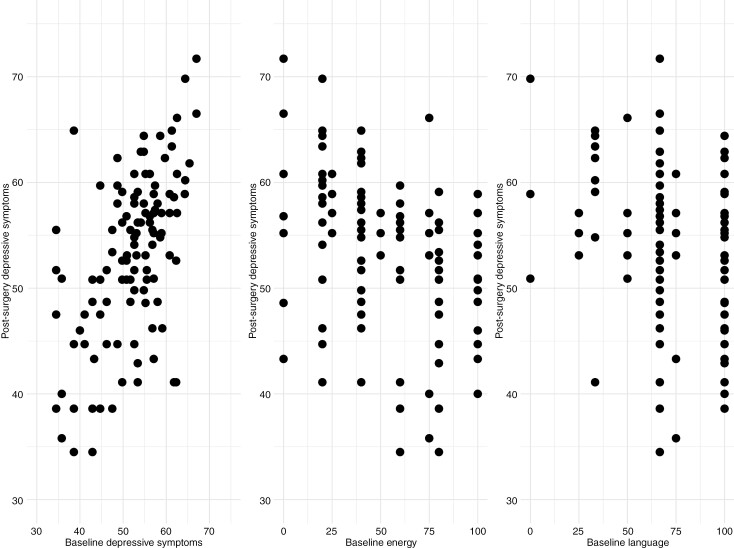
Scatter plots of postoperative depressive symptoms. Higher depressive symptom scores reflect more depressive symptoms. Higher ICF energy and language functions reflect better functioning.

A higher baseline depressive symptom score and lower ICF domain functions of energy and language were related to higher depressive symptom scores at follow-up in the final multivariable regression model (Table 2; R-squared 0.379). See Figure 2 for a scatter plot of these factors.

**Table 2.** Final Multivariable Regression Model for Depressive Symptoms at One Year Post-surgery

Term	Estimate	Standard error	*P-*value
(intercept)	36.982	5.945	<.001
Depressive symptoms	0.432	0.086	<.001
Energy	−0.048	0.022	.033
Language	−0.053	0.024	.031

The R-squared of the model is 0.379.

There were no strong correlations between factors (Supplementary Figure 2). As the LPA classes consist of the individual ICF domain factors, we repeated the stepwise backward regression without the LPA classes, which yielded identical results. Then, a best subset selection analysis confirmed depressive symptoms at baseline and ICF functions of language and energy to be robust factors (Supplementary Figure 3).

## Discussion

This study aimed to identify, through a multivariable model, the key patient and tumor characteristics—including ICF domain functions—at baseline that relate to depressive symptoms one year after surgery in patients with diffuse glioma. We found that baseline depressive symptoms and baseline lower energy and language functions, which are potentially modifiable, related to depressive symptoms 1 year after surgery.

Energy functions in the ICF encompass fatigue, which is one of the most severe and frequent symptoms in patients with diffuse glioma.^24–26^ Insomnia or hypersomnia are criteria that can be seen with major depressive disorder,^27^ and fatigue have been associated with depression in glioma, making effective treatment highly recommended.^2,26,28^ However, there is currently insufficient evidence for effective treatment of fatigue specifically in glioma patients.^29^ Nonetheless, we hope for positive results from a trial investigating cognitive behavioral therapy.^30^ For patients with cancer-related fatigue, interventions such as psychosocial and mind-body techniques might not only improve fatigue but if implemented early, could possibly also prevent its escalation.^31^

Language functions in the ICF encompass understanding and expression of language, and its dysfunctions are referred to by the term aphasia.^32^ Patients with aphasia after stroke are at risk for post-stroke depression,^33^ and even though pathophysiological mechanisms for language dysfunctions after stroke differ from those resulting from diffuse glioma, a shared correlation with depressive symptoms is plausible. A recent study supports the link between language dysfunction and mood disorders for diffuse glioma.^34^ Language dysfunction is common in glioma patients and language network reorganization may improve language function both pre- and postoperatively.^35^ The NICE guideline on neurorehabilitation in brain tumors suggests speech therapy based on expert opinion but evidence is lacking for the treatment of language dysfunction in brain tumor patients.^36^ Indeed, in post-stroke aphasia, various treatments such as behavioral speech and language therapy can improve language and treatments can possibly aid in reorganizing the language network.^37^ Initiating treatment early could be advantageous, or at the very least, not exacerbate aphasia.^38^

Depressive symptoms a year after surgery did not relate to the other patient- and tumor-related factors that we investigated. This corroborates the literature demonstrating no or weak associations between depression and patient- and tumor-related factors except for physical function and cognition.^6^

Baseline depressive symptoms strongly correlated with lower family functions. Family functioning has been demonstrated to be consistently impaired during disease, as reported by patients and caregivers.^14^ The correlation between baseline depressive symptoms and family function could be explained by the substantial symptom burden straining social connections and relationships.^39^ This strong correlation between baseline depressive symptoms and family function reflects that family function might be important for the early onset of depressive symptoms, whereas for depression a year post-surgery this effect is overtaken by baseline depressive symptoms, language, and energy functions.

We specifically assessed baseline characteristics to identify risk factors for depressive symptoms a year after surgery. We chose this timeframe for potential early anticipation of ensuing mood dysregulation with risk factors that are usually available for these patients. However, during the disease, depressive symptoms might also be affected by other functioning impairments or symptoms. Pain, for example, is infrequent in the newly diagnosed stage but is associated with depression in patients with cancer.^24,25,40^ Moreover, depressive symptoms do not necessarily reflect major depressive disorder and the etiology might be different from the general population.

Depressive symptoms in glioma patients are likely multifactorial, arising from a combination of interacting factors. While we assessed a broad range of patient and tumor characteristics and patterns of functioning according to the ICF domains, we did not evaluate all possible factors within this biopsychosocial model. Neurobiological factors, such as local tumor effects or systemic inflammation, were underrepresented but could also contribute to depressive symptoms.^41,42^ Additional psychological factors, including preexisting vulnerabilities like coping styles or previous depressive episodes, possibly also play a role.^5,6,43,44^ Moreover, although we did incorporate some social factors such as family functions, a more detailed assessment would be beneficial, as a good support system or relationship status might protect against the development of depressive symptoms.^44^

### ICF Domains

The ICF framework is based on a biopsychosocial model and creates a transdiagnostic framework to quantify functions important in (neuro) rehabilitation. We included the ICF domains based on the items of widely used questionnaires in brain tumor patients. This resulted in the inclusion of fifteen items that are considered important in newly diagnosed glioma patients. Drowsiness, sleeping problems, cognitive deficits other than language functions and seizures were not included,^25,45^ because the items were not included in both the FACT-Br and BN20 or SF-36, or because the underlying items could not be related to the ICF domains, but reflected symptoms rather than functional impact. For instance, the functional impact of seizures can vary between individuals with similar seizure frequency.^46^ Also, we were unable to include environment functions as these were not part of the questionnaires. Nevertheless, these environmental factors, such as family and social support or network, physical surroundings, or cultural beliefs and norms might be important for coping with impairments and for reducing depressive symptoms.^47,48^ These environmental factors may contribute to depressive symptoms a year after surgery and are of interest to explore in future studies.

We included both individual ICF domains and latent profile classes. The individual functions language and energy were significantly related to depressive symptoms a year post-surgery, whereas the latent patient profiles were not. However, some of these individual functions were moderately correlated. In previous symptom network analyses, language converged to a cognitive cluster and fatigue to a physical cluster or a separate cluster.^25,28^ Perhaps, a network approach with a much larger sample size rather than using multiple individual functions may prove beneficial to better understand long-term depressive symptoms, as individual factors may cooccur or affect each other.^49^

### Strengths and Limitations

The strengths of this study are that we pooled data from 2 cohorts to increase the external validity of our findings. Furthermore, we focused on potential risk factors at baseline which are usually available for patients, and we prespecified the timing of follow-up to improve clinical relevance. Additionally, we applied the ICF framework and therein explored the relevance of self-reported functional status items to enable comparison with other populations using other instruments with other items. We also applied rigorous complete case analyses and verified robustness.

The limitations of this study are that some characteristics of the 2 cohorts differed, possibly due to different patient selection and case referrals. Furthermore, larger (combined) cohorts could yield even more reliable results, increasing generalizability. Also, the ICF domains were linked on a conceptual basis rather than quantified. Nevertheless, this framework enables comparison of measurements in cohorts that apply different, but conceptually comparable instruments, which has been well-described.^11,12^ Moreover, the IRT conversion has not been validated in brain tumor patients specifically.^18,19^ Additionally, we included patients who were generally performing well, with most being fully active, indicating a likely selection bias. Finally, although we did include many patient-, treatment-, and functioning-related factors, we did not include all variables that could contribute to depressive status such as prior depression history, use of medication such as antidepressants, dexamethasone, or antiseizure drugs, and the presence of epilepsy. Future research could explore these factors, potentially enhancing the model’s performance and increasing the explained variance of depressive symptoms. Additionally, examining change scores might provide further insights, particularly when considering the minimal clinically important difference.

## Conclusions

In patients with diffuse glioma, more depressive symptoms, lower ICF energy function, and worse ICF language functions at baseline are associated with more depressive symptoms at 1-year post-surgery. Other patient and tumor characteristics did not add to the model. These risk factors may be relevant to identify those at risk early for patient education, and timely interventions.

## Supplementary Material

npae085_suppl_Supplementary_Table_S1_Figures_S1-S3
