# Supplementary material for: Baseline factors relating to depressive symptoms at one year postoperative in patients with diffuse glioma
Source: Neurooncol Pract. 2024 Sep 20;12(1):122–30. doi: 10.1093/nop/npae085 (PMC11798611; doi:10.1093/nop/npae085)
Supplement: npae085_suppl_Supplementary_Table_S1_Figures_S1-S3 [file npae085_suppl_supplementary_table_s1_figures_s1-s3.docx]

Supplementary material to:

Baseline factors relating to depressive symptoms at one year postoperative in patients with diffuse glioma

Vera Belgers^a^, Anders Tolver, Martin Klein, Linda Douw, Johanna M. Niers, Karin Piil* and Philip C. de Witt Hamer*

^a^Correspondence to Vera Belgers, Amsterdam UMC location Vrije Universiteit Amsterdam, Neurology, De Boelelaan 1117, Amsterdam, The Netherlands, [v.belgers@amsterdamumc.nl](mailto:v.belgers@amsterdamumc.nl)
*These authors share last authorship

Neuro-Oncology Practice

## ICF classification and domains

For creating the ICF domains, the most specific ICF domain was included. For instance, when items were linked to the general category “B156: Perceptual functions” in their paper, but a more specific subcategory was available in their provided data, such as “B1561: Visual perception” we linked to the more specific subcategory. When multiple items in a questionnaire linked to an ICF domain, such as “double vision” and “blurred vision” to “B1561: Visual perception”, we averaged them if they corresponded to similar items in the other instrument. We prioritized harmonization: if multiple items in an instrument referred to the same underlying construct, but only one of these items aligned with the item in the other questionnaire, we only linked that item (e.g. “Did you have trouble with coordination” was linked to “I have trouble with coordination”, and the item that also referred to “B760: Control of voluntary movement functions” was omitted). Each questionnaire item could only be linked to one underlying ICF domain to avoid overlap between ICF functioning domains.

After matching the items to the ICF domains, we omitted the ICF domain B260 proprioceptive functions as its matched items were also used in D415 and B840. This resulted in fifteen individual ICF domains: B130 energy and drive functions (“energy”), B1561 visual perception (“vision”), B167 mental functions of language (“language”), B28010 pain in head and neck (“headache”), B280 sensations of pain (“pain”), B370 muscle power functions (“motor function”), B760 control of voluntary movement functions (“coordination”), B840 sensation related to skin (“sensation”), D166 reading (“reading”), D330 speaking (“speaking”), D415 maintaining a body position (“body position”), D540 dressing (“dressing”), D760 family relationships (“family”), D850 remunerative employment (“work”) and D920 recreation and leisure (“recreation”). See Fig. 1 in the main text for the distribution of the ICF domains.

**Table 1:** Explorative univariable regressions of possible risk factors for depressive symptoms at one year post-surgery

| Factor | Term | Estimate | std. error | Statistic | *p-*value |
| --- | --- | --- | --- | --- | --- |
| Age |  | 0.037 | 0.051 | 0.739 | 0.461 |
| Sex (Reference: Male) | Female | -0.181 | 1.523 | -0.119 | 0.906 |
| Education level (Reference: High) | Medium | 4.106 | 1.673 | 2.454 | 0.016* |
|  | Low | 2.802 | 1.797 | 1.560 | 0.122 |
| Performance status (Reference: Fully active) | Minor restrictions | 4.384 | 1.564 | 2.802 | 0.006* |
|  | Limited activity | 6.701 | 2.999 | 2.234 | 0.027* |
| Tumor location (Reference: Frontal) | Non-frontal | -1.402 | 1.456 | -0.963 | 0.338 |
| Tumor side (Reference: Left) | Right | 2.681 | 1.469 | 1.826 | 0.071 |
|  | Bilateral | 5.198 | 4.029 | 1.290 | 0.2 |
| Histology (Reference: Astrocytoma) | Oligodendroglioma | 1.831 | 1.745 | 1.049 | 0.296 |
|  | Glioblastoma | 1.791 | 1.823 | 0.983 | 0.328 |
|  | Other | 1.288 | 8.034 | 0.160 | 0.873 |
| Grade (Reference: II) | III | -1.056 | 1.827 | -0.578 | 0.564 |
|  | IV | 0.526 | 1.722 | 0.305 | 0.761 |
| Baseline depressive symptoms |  | 0.566 | 0.075 | 7.556 | <0.001** |
| Energy |  | -0.105 | 0.023 | -4.640 | <0.001** |
| Vision |  | -0.055 | 0.031 | -1.790 | 0.076 |
| Language |  | -0.104 | 0.027 | -3.780 | <0.001** |
| Headache |  | -0.039 | 0.026 | -1.544 | 0.125 |
| Pain |  | -0.072 | 0.030 | -2.396 | 0.018* |
| Motor function |  | -0.064 | 0.032 | -2.016 | 0.046* |
| Coordination |  | -0.092 | 0.026 | -3.483 | <0.001** |
| Sensation |  | -0.089 | 0.032 | -2.790 | 0.006* |
| Reading |  | -0.081 | 0.027 | -3.005 | 0.003* |
| Speaking |  | -0.055 | 0.031 | -1.751 | 0.083 |
| Body position |  | -0.080 | 0.029 | -2.792 | 0.006* |
| Dressing |  | -0.026 | 0.037 | -0.714 | 0.477 |
| Family |  | -0.065 | 0.019 | -3.360 | 0.001* |
| Work |  | -0.070 | 0.017 | -4.051 | <0.001** |
| Recreation |  | -0.076 | 0.021 | -3.638 | <0.001** |
| LPA class (Reference: High) | Moderate | 4.230 | 1.498 | 2.824 | 0.006* |
|  | Low | 7.400 | 2.002 | 3.696 | <0.001** |

**p*-value <0.05; ** *p*-value <0.001


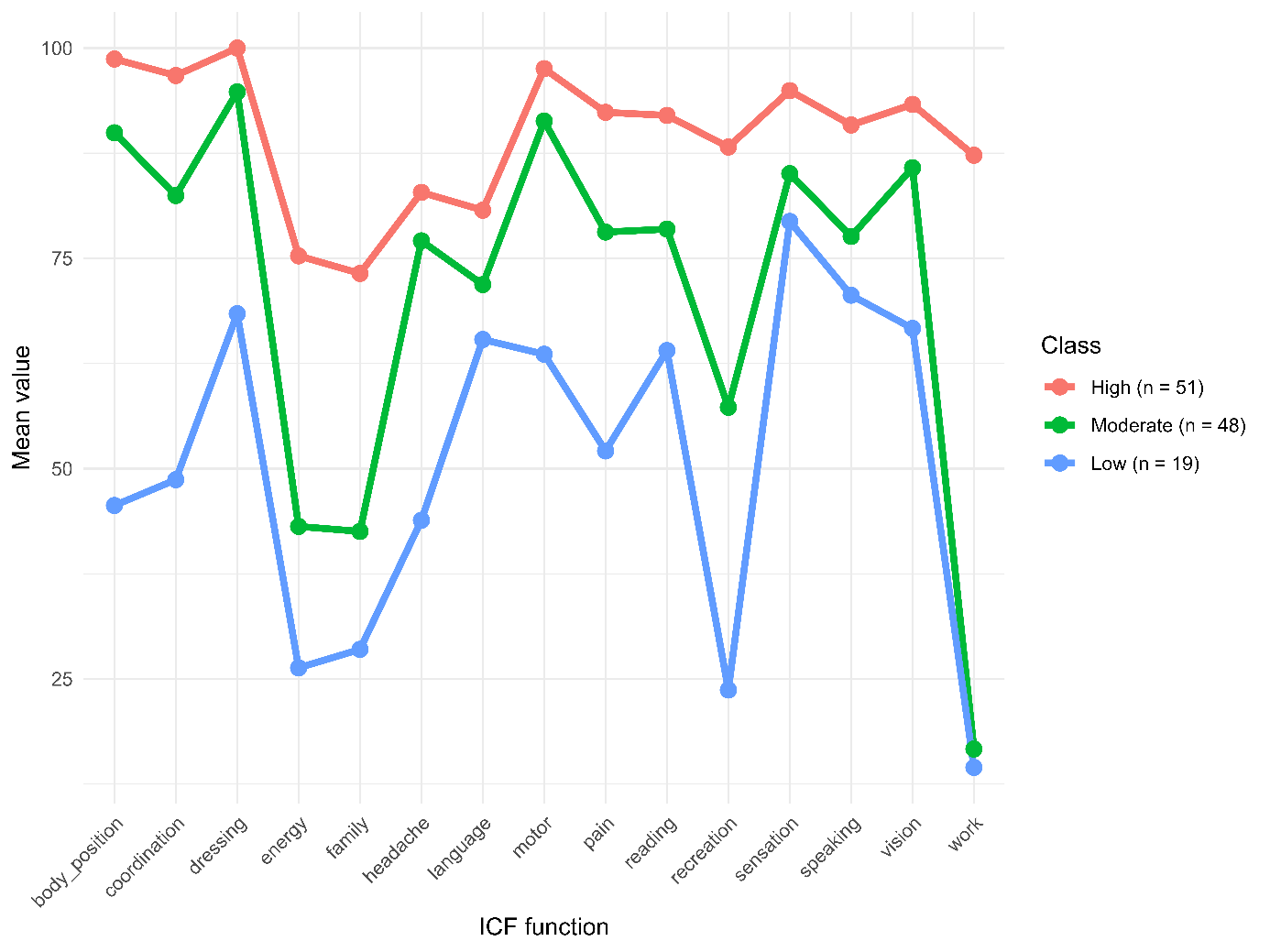


**Fig. 1** Mean ICF domains for each LPA class. We chose an optimal LPA model based on the Bayes information criterion (BIC), using the R package “mclust”.^1^ The LPA showed the optimal model to have equal volumes and shapes of the covariance matrix, and the optimal number of classes was three.^2^ We labeled the classes “high”, “moderate” and “low”, reflecting the overall ICF functions of patients belonging to a class.


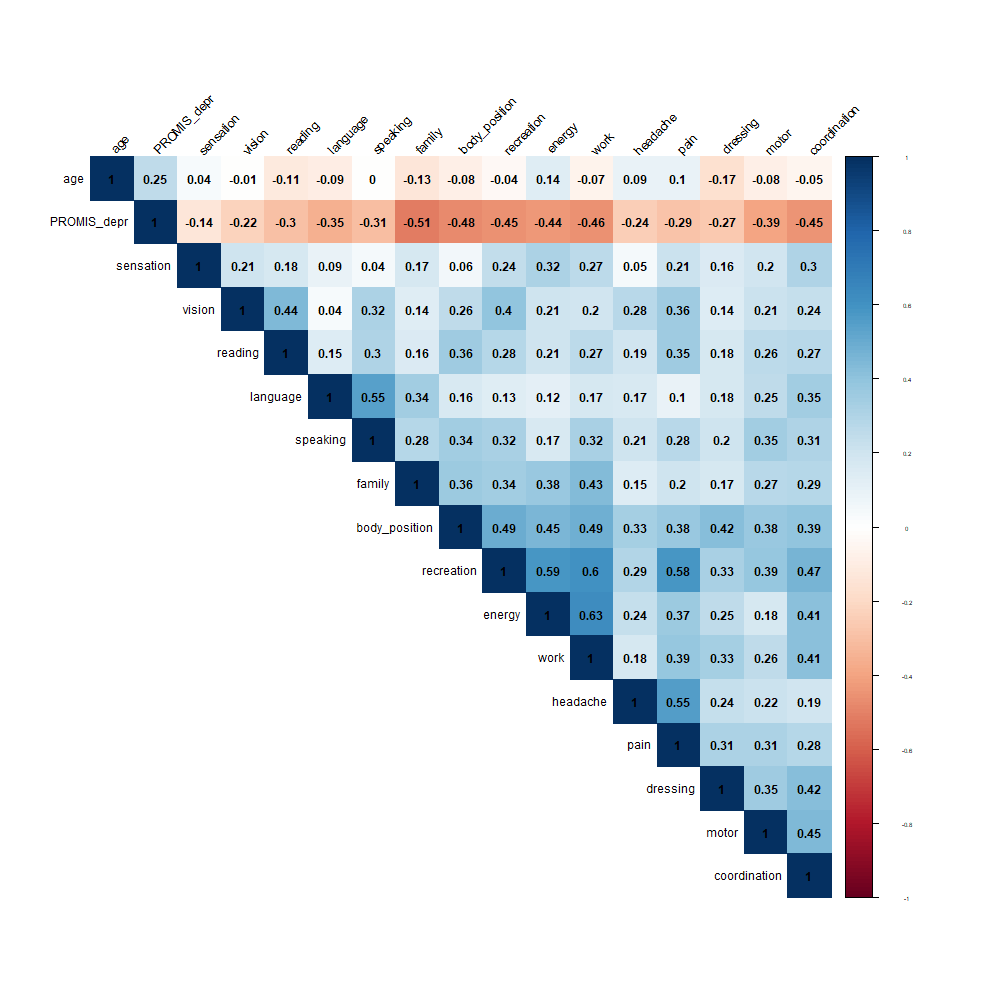
 **Fig. 2** Matrix of Spearman’s correlation coefficients for baseline continuous factors





**Fig. 3 Best subset selection.** The x-axis show the different factors included in the models, the y-axis shows the (non-adjusted) R-squared. The dark blocks represent which variables are included in the best subset analysis. A row with only two blocks represents the model with one variable (intercept and the best univariable factor), the row with three blocks represents the best subset model with two variables etcetera.

**References**

1. Scrucca L, Fraley C, Murphy T, Raftery A. Model-Based Clustering, Classification, and Density Estimation Using mclust in R. In: *Chapman and Hall/CRC*; 2023. doi:10.1201/9781003277965

2. Scrucca L, Fop M, Murphy TB, Raftery A. mclust 5: Clustering, Classification and Density Estimation Using Gaussian Finite Mixture Models. *R J*. 2016;8(1):289-317.
